# Prenatal diagnosis and management of Milroy syndrome: a case report

**DOI:** 10.1515/crpm-2023-0013

**Published:** 2023-09-07

**Authors:** Zacharias Fasoulakis, Marianna Chatziioannou, Antonios Koutras, Marianna Theodora, Afroditi Pegkou, Andreas Pampanos, George Daskalakis, Panagiotis Antsaklis

**Affiliations:** 1st Department of Obstetrics and Gynecology, National and Kapodistrian University of Athens, Athens, Greece; Department of Genetics, “General Hospital Alexandra”, Athens, Greece

**Keywords:** Milroy syndrome, prenatal diagnosis, limb edema, prenatal screening

## Abstract

**Objectives:**

Milroy syndrome is a rare hereditary disorder characterized by congenital lymphedema, caused by mutations in the vascular endothelial growth factor receptor 3 (*VEGFR3*) gene.

**Case presentation:**

We present a case report of a first-described mutation of a male fetus diagnosed prenatally with Milroy syndrome through amniocentesis. The fetus had bilateral lower limb edema, and genetic testing confirmed the diagnosis of Milroy syndrome. The patient was closely monitored throughout the pregnancy, and after delivery, the infant was managed with appropriate therapies, including compression garments and manual lymphatic drainage. The parents were provided with appropriate counseling and support.

**Conclusions:**

This case highlights the significance of early detection and appropriate management of Milroy syndrome, which can lead to improved outcomes for affected infants.

## Introduction

Milroy syndrome (MS) or primary congenital lymphedema is a type of hereditary primary lymphedema and is the most common form of this group of diseases [1, 2]. The syndrome is named after William Milroy, who was the first to study and describe almost one hundred members of a family in the 19th century, of whom almost 1/3 had leg edema [3]. The main characteristic of MS is lower-limb lymphedema, with an incidence of 1 in 33,000 deliveries and a 1:2 male/female ratio. It is inherited in an autosomal dominant pattern, with penetrance ranging from 80 to 100 % [1], [2], [3], [4]. The *FTL4* gene, which encodes vascular endothelial growth factor receptor 3 (*VEGFR 3*), represents currently the only known gene associated with MS [3, 5, 6]. Here, we present a rare case of a prenatally confirmed diagnosis of MS, due to a c.3116C>A p. (Ala1039Asp) unknown mutation in *the FLT4* gene.

## Case presentation

A 31-year-old pregnant woman who had previously undergone a Cesarean section was referred to our department‘s ultrasound unit for a second-trimester morphological ultrasound screening. The patient had an unremarkable family history and did not use any drugs and without previous exposure to teratogens during the first trimester of pregnancy. Serological tests for various infectious diseases were negative, and a previous ultrasound exam at 14 weeks was normal. The triple test for fetal abnormalities was also negative at 16 weeks.

**Figure 1:**
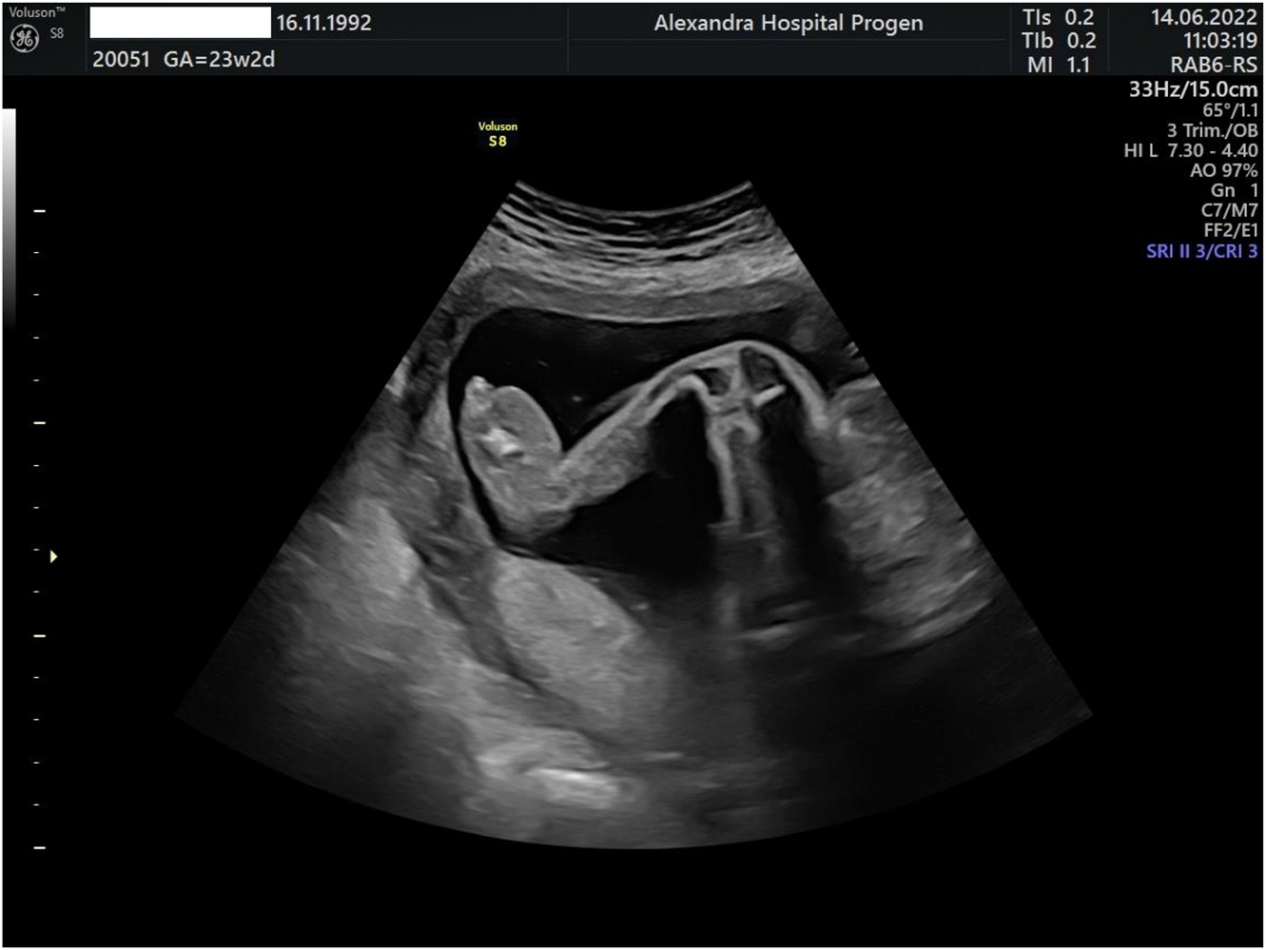
2D image of lower limp edema.

During the 20th-week ultrasound scan, a male singleton fetus with a composite sonographic age of 20 weeks and 2 days was observed to have significant edema of the lower limbs, and normal amniotic fluid (Figures 1 [Fig j_crpm-2023-0013_fig_002]–3). A follow-up exam in 2 weeks was recommended, along with tests for TORCH and indirect Coombs testing.

**Figure 2: j_crpm-2023-0013_fig_002:**
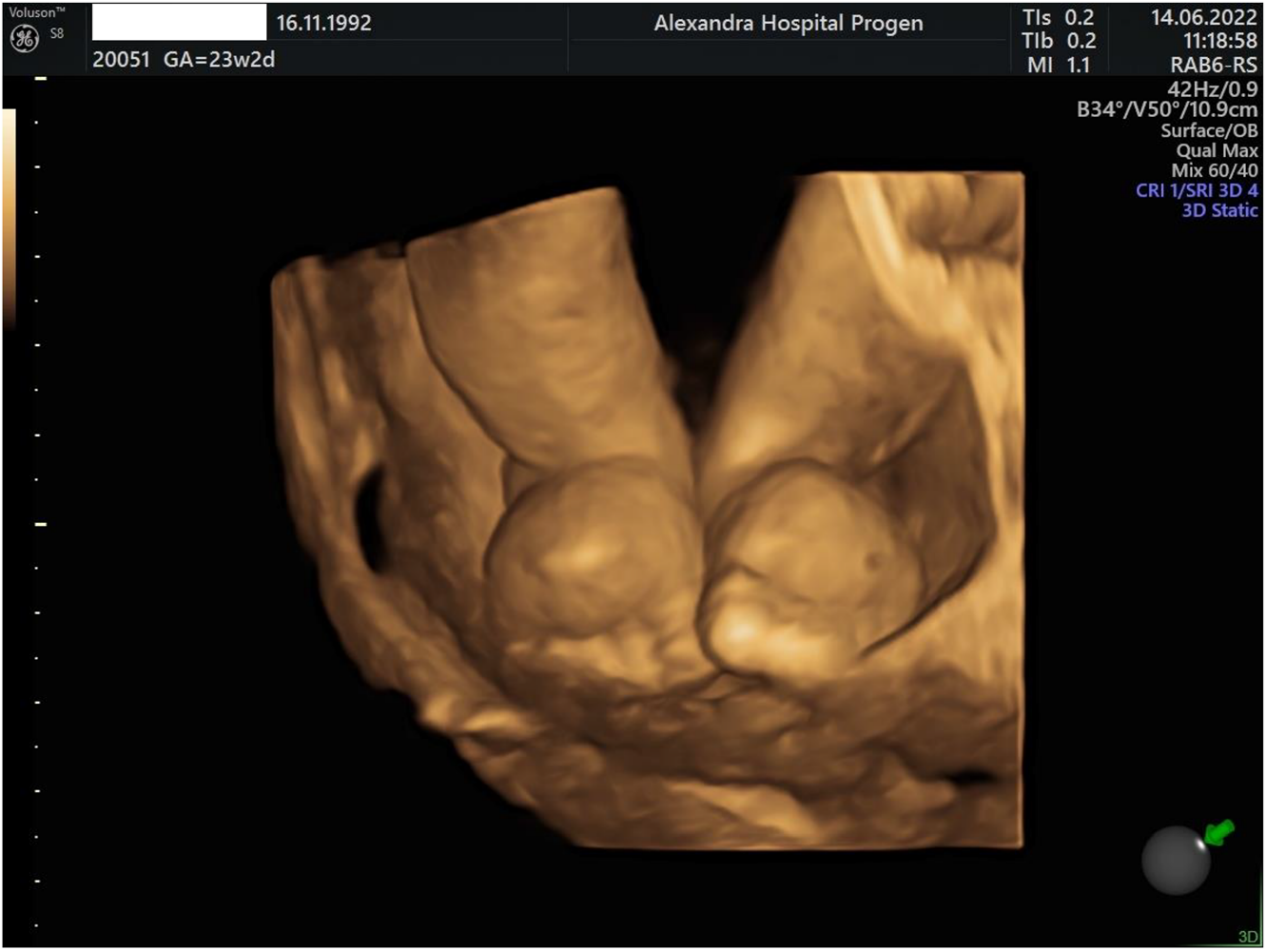
3D image of lower limp edema.

**Figure 3:**
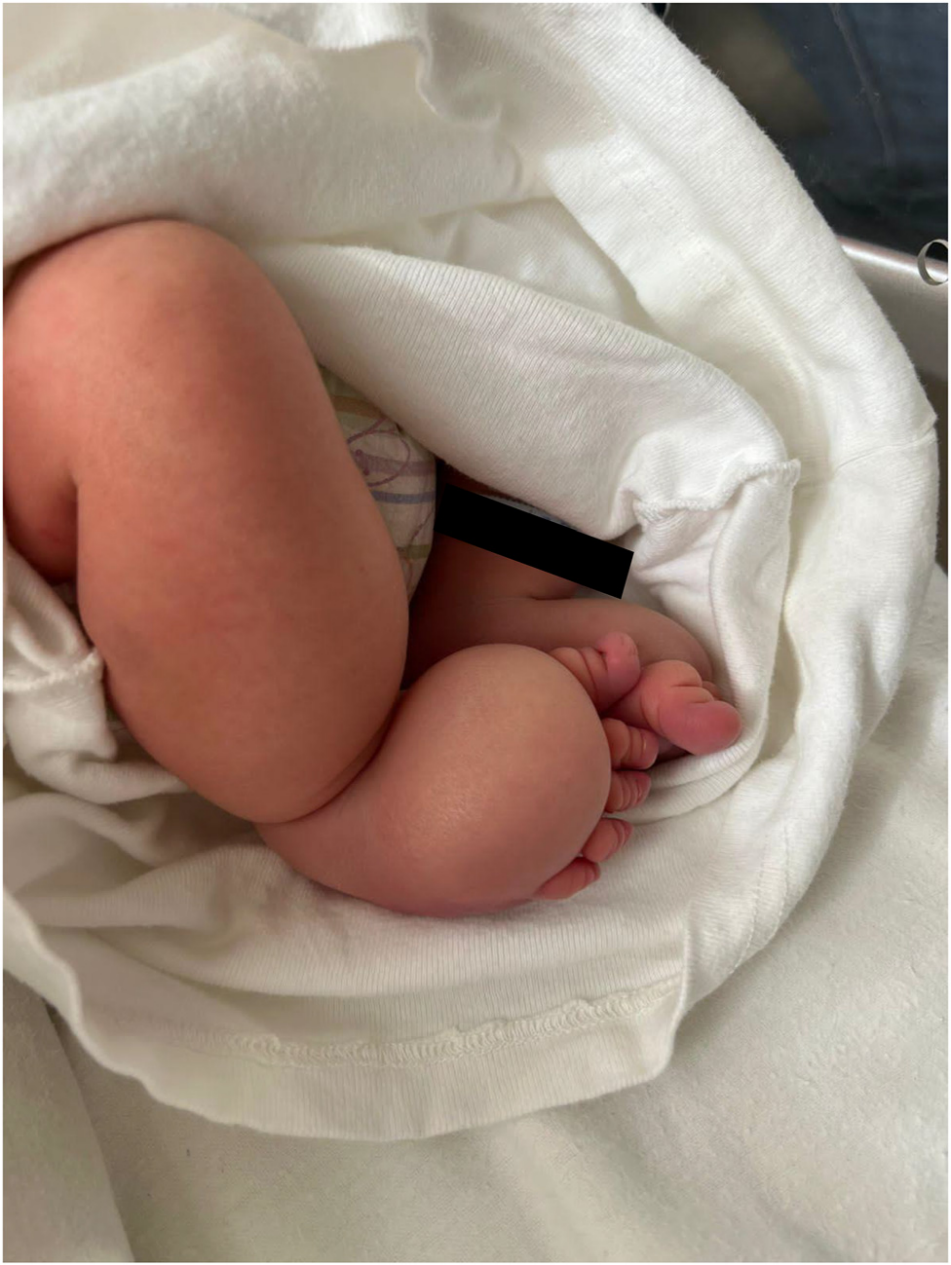
Lower limb edema on both legs recognized postpartum.

However subcutaneous edema on both lower limbs was again observed during the 23-week morphology ultrasound scan, suggesting a possible diagnosis of MS (Figures 1 and 2). Due to the persisted lymphedema in the lower limbs an amniocentesis was performed.

Subsequent testing revealed that the male karyotype was normal (46, XY), and molecular analysis for Noonan‘s syndrome was negative. Nucleotide sequencing of the amniotic fluid was performed with Illumina sequencing by 150 bases. Bioinformatics system was applied ((North-Oehler Aligarh, GATX) while additionally checking for the existence of deletions/duplications/CNVs) (Copy Number Variations) in the *FLT4* gene was performed. The analysis identified a c.3116C>A p. (Ala1039Asp) mutation, that is a not known pathogenic variant in the *FLT4* gene, confirming the diagnosis of hereditary lymphedema type 1A – Milroy disease. Maternal blood analysis confirmed maternal mutation and genetic counseling followed.

Cesarean section was performed at 38 weeks and 6 days of gestational age, and a male neonate weighing 3,850 g and measuring 46 cm with an Apgar score of 9/10 was born. Both lower limb edema was recognized after delivery (Figure 3). Histopathological examination of the placenta, umbilical cord, and amniotic sac showed no signs of chorionamnionitis or other pathology.

## Discussion

MS represents a genetic disorder with the *FLT4* gene playing a critical role in the development and maintenance of the lymphatic system [5]. Mutations in the *FLT4* gene lead to the disruption of *VEGFR-3* signaling, resulting in the malformation and dysfunction of the lymphatic vessels [6]. This subsequently leads to impaired lymphatic drainage and accumulation of lymphatic fluid in the affected tissues, causing lymphedema [7].

Recent studies have suggested that the severity of MS is determined by the type of *FLT4* gene mutation and its impact on *VEGFR-3* function [8]. Some mutations result in a complete loss of *VEGFR-3* function, leading to severe cases of lymphedema, while other mutations only partially impair the receptor’s function, resulting in milder cases [9].

The early detection of MS is essential for the effective management of the condition and prevention of complications. Prenatal diagnosis can provide families with valuable information to make informed decisions about the pregnancy and prepare for the potential challenges associated with the disorder. The prenatal diagnosis of MS typically involves a combination of imaging studies and genetic testing [10].

Ultrasound is the most commonly used imaging modality for prenatal diagnosis, as it is non-invasive and safe for both the mother and the fetus [11]. Fetal ultrasound can detect signs of lymphedema, such as limb swelling and skin thickening, as early as the second trimester [12]. However, these findings can be non-specific and might be associated with other conditions, necessitating further investigation.

Confirmatory diagnosis of MS is based on the identification of a pathogenic mutation in the *FLT4* gene through genetic testing [10]. Prenatal genetic testing can be performed using different methods, such as chorionic villus sampling (CVS) or amniocentesis. CVS is typically performed between 10 and 13 weeks of gestation and involves the collection of placental tissue for genetic analysis [13]. As mentioned by previous studies, amniocentesis is the usualy diagnostic test performed around the 20th week of gestation for fetal genetic analysis, since the main ultrasound findings are not visual at first-trimester ultrasound screening [14], [15], [16]. Both methods carry a small risk of miscarriage, and the choice of method depends on the gestational age and individual risk factors [17].

Recently, other hereditary mutations were also described. Lajmi et al. reported two novel genetic variations p.(Ser1275Thr) and p.(Ser1275Arg) in the *FLT4* gene, which they discovered were responsible for causing prenatal hereditary lymphedema type 1, a rare genetic disorder marked by swelling in the arms and legs due to a blockage in the lymphatic system. These variants were identified through extensive genetic analysis of affected individuals, highlighting the role of the *FLT4* gene in the development of the lymphatic system and the pathogenesis of hereditary lymphedema [15]. In addition, Huynh and colleagues have identified a connection between a heterozygous deletion of the *VEGFC* gene in the 4q34.3 region of the chromosome and a Milroy-like lymphedema condition. This discovery resulted from the investigation of an unprecedented prenatal case, thereby providing crucial insights into the genetic underpinnings of this type of lymphedema. The genetic deletion was detected in a prenatal context, marking the first instance of such an early diagnosis. This finding implies that the identified VEGFC gene deletion could serve as a significant genetic marker, potentially allowing for earlier detection, improved management, and potential intervention strategies for the condition, even in a prenatal scenario [16].

There is currently no cure for MS; however, appropriate management can significantly improve the quality of life and prevent complications. Appropriate management of MS can significantly improve the quality of life and prevent complications. The management of MS involves a multidisciplinary approach, including physical therapy, compression garments, skin care, and psychological support.

The primary goal of physical therapy is to reduce swelling, improve range of motion, and enhance overall limb function. Manual lymphatic drainage (MLD), a specialized massage technique, is often employed to stimulate the flow of lymphatic fluid and reduce edema [18]. Exercise programs tailored to the individual’s needs and abilities can also help improve muscle strength, flexibility, and endurance [19].

Custom-fitted compression garments, such as stockings or sleeves, can help maintain the reduction in limb volume achieved through physical therapy and MLD [20]. These garments apply gradual pressure to the affected limb, encouraging the flow of lymphatic fluid and preventing its accumulation [21].

Proper skin care is crucial in the management of MS, as the risk of infection is increased due to impaired lymphatic function. Patients should be advised to maintain good hygiene, moisturize the skin regularly, and avoid cuts or injuries to the affected limb [22].

Psychological support is another issue of great importance. Living with a chronic condition such as MS can have a significant impact on mental health and well-being. Access to psychological support services, such as counseling or support groups, can help individuals and their families cope with the emotional challenges associated with MS [23].

## Conclusions

Milroy syndrome is a rare congenital lymphatic disorder that can significantly impact the quality of life for those affected. Early diagnosis, including prenatal detection, is crucial for the effective management of the condition and prevention of complications. A multidisciplinary approach, involving physical therapy, compression garments, skin care, and psychological support, is essential for the management of MS. Further research is needed to explore novel therapeutic approaches and improve the understanding of the pathophysiology of this rare disorder.
